# Is There any Difference Between the Glomerular Filtration Rate of Patients With Chronic Hepatitis B and C and Patients With Cirrhosis?

**DOI:** 10.5812/hepatmon.6789

**Published:** 2013-04-15

**Authors:** Cristina Gluhovschi, Silvia Velciov, Roxana Buzas, Ligia Petrica, Gheorghe Bozdog, Florica Gadalean, Adrian Gluhovschi, Cristian Balgradean, Corina Vernic, Ioan Sporea

**Affiliations:** 1 Division of Nephrology, University of Medicine and Pharmacy (V. Babes), Timisoara, Romania; 2 Emergency County Hospital, University of Medicine and Pharmacy (V. Babes), Timisoara, Romania; 3 Department of Medical Informatics and Biostatistics, University of Medicine and Pharmacy (V. Babes), Timisoara, Romania; 4 Division of Gastroenterology and Hepatology, University of Medicine and Pharmacy (V. Babes), Timisoara, Romania

**Keywords:** Hepatitis B Virus, Hepatitis C, Liver Cirrhosis, Glomerular Filtration Rate

## Abstract

**Background:**

Renal dysfunction is a major determinant of the Model of End-stage Liver Disease (MELD) score. The implementation of the MELD score has shifted allocation of livers to patients with renal dysfunction.

**Objectives:**

The aim of our study was the assessment of estimated Glomerular Filtration Rate (eGFR) by the Modification of Diet in Renal Disease 4 (MDRD4) method in patients with HBV chronic hepatitis, HCV chronic hepatitis, and cirrhosis (CH) caused by these viruses to detect any differences in renal function among these diseases.

**Patients and Methods:**

We performed a cross-sectional analysis of all consecutive patients with HBV chronic hepatitis, HCV chronic hepatitis, and cirrhosis caused by these viruses hospitalized during a 4 year period in the Gastroenterology and Hepatology department of the Emergency County Hospital Timisoara, Romania. The eGFR was assessed by the MDRD4 method. Statistical analysis (unpaired t-test, ANOVA, Chi Square test) was performed using OpenEpi 2.3.1.

**Results:**

HBV chronic hepatitis, HCV chronic hepatitis, and cirrhosis secondary to these viruses were associated with a reduction of the GFR. The eGFR was higher in patients with HBV chronic hepatitis than in patients with HCV chronic hepatitis (P &lt; 0.001). Patients with cirrhosis secondary to HBV infection had a higher eGFR than patients with cirrhosis secondary to HCV (P = 0.01). The eGFR of patients with HCV chronic hepatitis was higher than the eGFR of patients with cirrhosis due to this virus (P &lt; 0.001).

**Conclusions:**

Functional renal impairment in diseases caused by HCV was more important than in diseases caused by HBV. The eGFR was statistically lower in cirrhosis secondary to HCV than in HCV chronic hepatitis.

## 1. Background

Renal dysfunction, both acute and chronic, is common in patients with end stage liver disease (ESLD). On 2/27/2002, the United Network of Organ Sharing has implemented the Model of End-stage Liver Disease (MELD) scoring system, designed to allocate organs to ESLD patients with high predicted waitlist mortality. Renal dysfunction (i.e. creatinine and requirement for renal replacement therapy) is a major determinant of the MELD score. The implementation of the MELD score has shifted the allocation of livers to patients with renal dysfunction. According to Proulx, assessing kidney function in patients with cirrhosis has to take into account the following:

• Creatinine (Cr) assays are subject to interference by chromogens, bilirubin being the major one

• There is decreased hepatic production of creatine

• The edematous state which complicates end-stage liver disease leads to large distribution of Cr in the body and lower serum Cr concentration

• Complications such as variceal bleeding, spontaneous bacterial peritonitis or sepsis lead to increased Cr tubular excretion (1).

Measuring kidney function reliably, noninvasively and reproducibly is an unmet goal (2). This is even more difficult in patients with comorbidities such as cirrhosis (3). Various estimating equations have been developed, as measuring Glomerular Filtration Rate (GFR) in all patients would be impractical (4). The Modification of Diet in Renal Disease (MDRD) formula to estimate the GFR is the most used of the existing formulas for providing an assessment of kidney function which corresponds to the actual measurement of the GFR (5). Despite its limitations in patients with cirrhosis (6), serum creatinine is universally used to assess renal function in clinical practice and as part of the MELD score for prioritization of recipients for liver transplantation (6).

## 2. Objectives

The aim of our study was the assessment of estimated Glomerular Filtration Rate (eGFR) by the MDRD4 method in patients with HBV chronic hepatitis, HCV chronic hepatitis, and cirrhosis (CH) caused by these viruses to detect any differences in renal function among these diseases.

## 3. Patients and Methods

This was an observational cross-sectional study performed on patients with HBV chronic hepatitis, HCV chronic hepatitis, and cirrhosis caused by these viruses hospitalized during a 4 year period in the Gastroenterology and Hepatology department of the Emergency County Hospital Timisoara, Romania. All consecutive patients hospitalized during a 4 year period with a diagnosis of HBV chronic hepatitis, HCV chronic hepatitis, cirrhosis secondary to HBV infection, and cirrhosis secondary to HCV infection were included in the study. We identified 1162 patients. We decided to exclude 122 patients with confounding factors as follows: 24 patients with HBV and HDV infection, 21 patients with HBV and HCV infection, 1 patient with HBV+HCV+HDV infection, 5 patients in whom alcohol consumption was superimposed on HCV infection, 3 patients in whom alcohol consumption was superimposed on HBV infection, 1 patient with hemochromatosis and HBV infection, 21 patients in whom alcohol consumption was superimposed on cirrhosis secondary to HBV infection, 15 patients in whom alcohol consumption was superimposed on cirrhosis secondary to HCV infection, 17 patients with cirrhosis secondary to HBV and HDV infection, 12 patients with cirrhosis secondary to HBV and HCV infection, 1 patient with cardiac cirrhosis superimposed on cirrhosis secondary to HBV and HCV infection, and 1 patient with hepatocellular carcinoma superimposed on cirrhosis secondary to HBV and HCV infection. Serum creatinine was measured by a modified Jaffe reaction (rate-blanked with compensation) on a HITACHI 717 analyzer. The estimated GFR (eGFR) was evaluated by the MDRD4 method. The study protocol conforms to the ethical guidelines of the 1975 Declaration of Helsinki, and was approved by the Ethics Committee of the Emergency County Hospital Timisoara Romania.

### 3.1. Statistical Analysis

Data are expressed as means ± SD. Comparison of the eGFR among the groups was performed with the unpaired t-test. Comparison of age among the groups was performed with ANOVA. Comparison of gender distribution and relative frequency of diabetes among the groups was performed with the Chi Square test. The statistical software used was OpenEpi 2.3.1. A P < 0.05 was considered as statistically significant.

## 4. Results

After the exclusion of 122 patients with confounding factors, 1040 patients with HBV chronic hepatitis, HCV chronic hepatitis, and cirrhosis caused by these viruses were identified. Two-hundred-three patients, 129 M, 74 F, mean age: 42.08 ± 12.96 years, presented with HBV chronic hepatitis. Five-hundred-ninety-one patients, 220 M, 371 F, mean age: 50.48 ± 11.17 years, presented with HCV chronic hepatitis. Seventy-six patients, 42 M, 34 F, mean age: 52.56 ± 10.52 years, presented with cirrhosis secondary to HBV infection (CH HBV). One hundred-seventy patients, 47 M, 123 F, mean age: 61.14 ± 10.93 years, presented with cirrhosis secondary to HCV infection (CH HCV). All patients were Caucasian. The eGFR of the patients is presented in Table 1.

**Table 1.** The Number, Age, Gender Distribution, Relative Frequency of Diabetes, and eGFR of the Patients With HBV Chronic Hepatitis, HCV Chronic Hepatitis, CH HBV, and CH HCV

Diagnosis	HBV^a^	HCV^a^	CH-HBV^a^	CH-HCV^a^
**No. of Patients**	203	591	76	170
**Age, y, Mean ± SD**	42.08 ± 12.96	50.48 ± 11.17	52.56 ± 10.52	61.14 ± 10.93
**Gender, No. (%)**				
Male	129 (63.54)	220 (37.22)	42 (55.26)	47 (27.64)
Female	74 (36.46)	371 (62.78)	34 (44.74)	123 (72.36)
**Relative Frequency of Diabetes, No. (%)**	24/203 (11.82)	94/591 (15.9)	8/76 (10.52)	49/170 (28.82)
**eGFR, ml/min/1.73 sqm**	83.39 ± 17.13	77.5 ± 16.07	79.5 ± 27.26	71.57 ± 22.81

^a^Abbreviations: CH-HBV, cirrhosis secondary to HBV; CH-HCV, cirrhosis secondary to HCV; HBV, hepatitis B virus; HCV, hepatitis C virus

• The eGFR was higher in patients with HBV chronic hepatitis than in patients with HCV chronic hepatitis (P < 0.001).

• Patients with cirrhosis secondary to HBV infection had a higher eGFR than patients with cirrhosis secondary to HCV (P = 0.01).

• The eGFR of patients with HCV chronic hepatitis was higher than the eGFR of patients with cirrhosis due to this virus (P < 0.001).

• The eGFR of patients with HBV chronic hepatitis was not statistically different from the eGFR of patients with cirrhosis secondary to this virus (P = 0.15).

The gender distribution among the diagnostic groups was varied (this was an observational study reflecting a real situation encountered in clinical practice). In Tables 2, 3, 4 and 5 we have shown the estimated GFR (eGFR) according to gender for patients with HBV chronic hepatitis, HCV chronic hepatitis, and cirrhosis caused by these viruses. Women had a lower eGFR than men within all 4 diagnostic groups (HBV chronic hepatitis, HCV chronic hepatitis, cirrhosis secondary to HBV, and cirrhosis secondary to HCV): P < 0.001; P < 0.001; P = 0.03; and P = 0.04 respectively. Of course, patients with HBV, HCV chronic hepatitis, and cirrhosis caused by these viruses can present many factors which could affect renal function, such as diabetes mellitus. The prevalence of diabetes mellitus was 11.82% (24/203) in HBV chronic hepatitis, 15.9% (94/591) in HCV chronic hepatitis, 10.52% (8/76) in cirrhosis secondary to HBV, and 28.82% (49/170) in cirrhosis secondary to HCV. An analysis of factors influencing renal function is beyond the scope of this paper. By comparing the age, gender distribution, and relative frequency of diabetes among the 4 diagnostic groups (HBV chronic hepatitis, HCV chronic hepatitis, cirrhosis secondary to HBV, and cirrhosis secondary to HCV) we found a statistically significant difference: P < 0.001 (for age by ANOVA), P < 0.001 (for gender distribution by Chi Square test), and P < 0.001 (for relative frequency of diabetes by Chi Square test). This is of course a limitation of our observational study, reflecting a real situation encountered in clinical practice. We consider, however, that as long as the MDRD4 formula for estimating GFR factors in age and gender, the differences in age and gender among the groups are accounted for when estimating GFR by the MDRD4 formula.

**Table 2.** The eGFR According to Gender for Patients With HBV Chronic Hepatitis

Gender	Mean ± SD	Patients, No.
**Female**	77.79 ± 16.65	74
**Male**	86.61 ± 16.63	129
**Total**	83.39 ± 17.13	203

**Table 3.** The eGFR According to Gender for Patients With HCV Chronic Hepatitis

Gender	Mean ± SD	No. of Patients
**Female**	73.33 ± 14.76	371
**Male**	84.54 ± 15.78	220
**Total**	77.50 ± 16.07	591

**Table 4.** The eGFR According to Gender for Patients With Cirrhosis Secondary to HBV Infection

Gender	Mean ± SD	No. of Patients
**Female**	71.93 ± 29.66	34
**Male**	85.63 ± 23.78	42
**Total**	79.50 ± 27.26	76

**Table 5.** The eGFR According to Gender for Patients With Cirrhosis Secondary to HCV Infection

Gender	Mean ± SD	No. of Patients
**Female**	69.42 ± 22.91	123
**Male**	77.17 ± 21.82	47
**Total**	71.57 ± 22.81	170

## 5. Discussion

Renal function in patients with cirrhosis is important prognostically, both before and following liver transplantation, as reflected by the inclusion of serum creatinine in the model for end-stage liver disease score (7). This had led to a prioritization of liver transplant allocation towards patients with renal dysfunction, and has reduced mortality among patients awaiting liver transplantation (3). Because renal function as assessed by serum creatinine has a major impact on access to a liver transplant, measurement of renal function in patients with cirrhosis is of paramount importance. In clinical practice, serum creatinine is still the most used method for assessing renal function in patients with cirrhosis. Although measurement of the glomerular filtration rate on the basis of the clearance of inulin and a variety of both „cold” and radioactive markers of kidney function represent the „gold standards”, they are impractical for routine clinical use (2). Measurement of creatinine clearance based on 24 h urine collections overestimates the GFR, and requires accurate urine collections, which is also not practical (2). Other biomarkers, such as cystatin C also appear to have errors (2-4). Although serum creatinine has been incorporated into the MELD score, it is known that a serum creatinine within the reference range does not exclude a significant impairment in the GFR (1). A number of different equations have been derived that incorporate serum creatinine to provide an estimation of the GFR: Cockroft-Gault (C-G), MDRD, and CKD-EPI. Both C-G and MDRD have limitations in patients with cirrhosis, and the utility of the CKD-EPI equation in patients with cirrhosis has not as yet been proven (3). These equations should never be employed in patients with acute kidney injury. Nevertheless, the MDRD4 formula is typically used in clinical practice for population screening. Despite its limitations in patients with cirrhosis, because serum creatinine within the normal reference range does not exclude a significant impairment in the GFR, there is a strong need in clinical practice to estimate the GFR in patients with cirrhosis who are not in an acute setting of renal function impairment. The aim of our study was to detect any differences in renal function in HBV chronic hepatitis, HCV chronic hepatitis, and cirrhosis caused by these viruses. We found that HBV chronic hepatitis, HCV chronic hepatitis, and cirrhosis secondary to these viruses were associated with a reduction of the GFR. The eGFR was higher in patients with HBV chronic hepatitis than in patients with HCV chronic hepatitis (P < 0.001). Patients with cirrhosis secondary to HBV infection had a higher eGFR than patients with cirrhosis secondary to HCV (P = 0.01). The eGFR of patients with HCV chronic hepatitis was higher than the eGFR of patients with cirrhosis due to this virus (P < 0.001). HBV and HCV chronic hepatitis are important causes of renal disease. At the same time, progression of HBV and HCV chronic hepatitis to cirrhosis could be accompanied by a decline in renal function. Therefore, an assessment of the GFR could offer a clue in this direction. There is a paucity of data in the literature regarding HBV infection and renal function, while data on renal function in HCV infection is conflicting. Kidney disease can have a negative impact on the natural history of HCV infection; patients with HCV and kidney disease often have adverse outcomes. Some authors, such as Asrani found no association between HCV and kidney disease (8), while Dalrymple reported that HCV was associated with an increased prevalence of renal insufficiency (9). Fabrizi et al. have recently performed a meta-analysis of published medical literature to determine whether HCV is associated with increased likelihood of kidney disease. They identified nine clinical studies. Pooling of study results demonstrated the absence of an association between HCV seropositive status and reduced estimated GFR (adjusted relative risk, 1.12; 95% confidence interval, 0.91, 1.38; P = 0.28) (10). Our study was performed on a large group of subjects. We found that HBV chronic hepatitis, HCV chronic hepatitis, and cirrhosis secondary to these viruses were associated with a reduction of the GFR, drawing attention to the importance of the assessment of renal function in patients with chronic hepatitis and cirrhosis. Renal function is an important predictor of survival in cirrhosis and liver transplantation (11). Serum creatinine is universally used in clinical practice to assess renal function (3), and estimated GFR can easily be computed; the MDRD4 formula being currently the most frequently used. As renal dysfunction is a challenging complication of cirrhosis and is one of the most important risk factors when liver transplantation is considered, attempts to estimate GFR from serum creatinine for screening purposes should be undertaken despite limitations in calculating equations (MDRD4 in our case) for patients who are not in an acute setting of renal dysfunction. Alternative methods for assessing renal function have been proposed. Despite promising results with the use of cystatin C, Xirouchakis stated in a recent paper that the estimated GFR in cirrhosis is not better with cystatin C formulas compared to creatinine ones (11). Inulin clearances are impracticable in routine clinical practice, as are single bolus isotopic and iodinated radiocontrast methods relying on timed urinary collections (7). Serial plasma measurements with delayed sampling would provide a more accurate estimate of the GFR, but are unlikely to be applied in routine clinical practice (3). HBV chronic hepatitis, HCV chronic hepatitis, and cirrhosis secondary to these viruses were associated with a reduction of the GFR. Renal function impairment in diseases caused by HCV was more important than in diseases caused by HBV. The eGFR was statistically lower in cirrhosis secondary to HCV than in HCV chronic hepatitis, which could signify that renal function impairment as assessed by the eGFR might parallel the severity of liver disease.
